# A high-resolution strain-gauge nanolaser

**DOI:** 10.1038/ncomms11569

**Published:** 2016-05-12

**Authors:** Jae-Hyuck Choi, You-Shin No, Jae-Pil So, Jung Min Lee, Kyoung-Ho Kim, Min-Soo Hwang, Soon-Hong Kwon, Hong-Gyu Park

**Affiliations:** 1 Department of Physics, Korea University, Seoul 136-701, Korea; 2 Department of Physics, Chung-Ang University, Seoul 156-756, Korea

## Abstract

Interest in mechanical compliance has been motivated by the development of flexible electronics and mechanosensors. In particular, studies and characterization of structural deformation at the fundamental scale can offer opportunities to improve the device sensitivity and spatiotemporal response; however, the development of precise measurement tools with the appropriate resolution remains a challenge. Here we report a flexible and stretchable photonic crystal nanolaser whose spectral and modal behaviours are sensitive to nanoscale structural alterations. Reversible spectral tuning of ∼26 nm in lasing wavelength, with a sub-nanometre resolution of less than ∼0.6 nm, is demonstrated in response to applied strain ranging from −10 to 12%. Instantaneous visualization of the sign of the strain is also characterized by exploring the structural and corresponding modal symmetry. Furthermore, our high-resolution strain-gauge nanolaser functions as a stable and deterministic strain-based pH sensor in an opto-fluidic system, which may be useful for further analysis of chemical/biological systems.

## Abstract (web summary)

Flexible, millimetre-scale systems and devices require embedded, reliable micrometre-scale components, which can be challenging to achieve for optical sensors. Here, the authors design a photonic crystal nanolaser and demonstrate its use in measuring strain.

## Graphical abstract

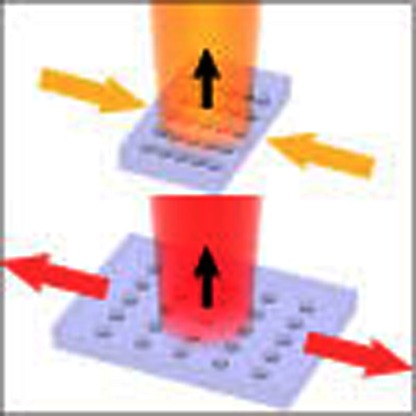

Strategies based on flexibility and stretchability for electronics have accelerated advancements in wearable and human-interactive electronics^1^,^2^,^3^,^4^,^5^,^6^,^7^,^8^,^9^,^10^,^11^. Various integration schemes for circuit elements involving organic/inorganic nanomaterial-based matrix arrays^1^,^2^,^3^,^4^,^5^,^6^,^7^,^8^,^9^,^10^, micro- and nanostructured hybrid composites^7^,^12^,^13^, and assemblies of nanowires or nanotubes^10^,^11^,^12^,^13^,^14^,^15^,^16^,^17^ have been developed to enable the measurement of the spatial distribution of input pressure/strain signals. These advancements have also motivated researchers to investigate structural/mechanical deformations at the fundamental scale because an understanding of structural changes at the submicron or nanometre scale can improve the device sensitivity and spatiotemporal response^8^,^9^,^14^,^15^,^16^,^17^. For example, sensors based on nanoscale crack junctions have demonstrated ultrahigh sensitivity under strain and vibration^16^. Shape-changing magnetizable micro-disk pairs have enabled spatiotemporal mapping of ion concentration gradients and co-localized sensing through spectrally separable sensors^17^. However, the mechanical assessment and characterization of micro- or nanoscale local alterations remain challenging for many flexible electronics because of limited measurement tools that require an overall device footprint on the micron scale, high precision with sub-nanometre resolution and high signal to noise contrast.

Nanolasers can be a powerful tool to address these limitations. Subwavelength- and wavelength-scale nanocavities can strongly confine electromagnetic energy within overall device footprints of a few micrometres or less without external complications^18^,^19^,^20^,^21^,^22^,^23^. A very sharp resonance behaviour with a sub-nanometre spectral resolution distinctively differentiates these nanolasers from other classes of active electrical and optical signal sources^18^,^19^,^20^,^21^,^22^,^23^. In addition, a large free spectral range between the resonant wavelengths in nanocavities often enables single-mode lasing, which can be easily monitored without confusion^22^,^23^. Furthermore, the spectral and modal behaviours of lasing modes are highly sensitive to the structural deformation of cavities^18^,^19^ and environmental changes such as changes in the refractive index^24^,^25^ or temperature^26^,^27^,^28^ of the surrounding medium. Among several nanolasers, photonic crystal (PhC) lasers are particularly interesting because they efficiently manipulate resonant lasing modes through rational design and engineering of photonic band structures for various purposes, such as resonant wavelength shifting and modal symmetry modification^18^,^19^,^23^,^24^. In this work, we demonstrate a flexible and stretchable PhC nanolaser, which is sensitive to nanoscale structural changes and thus can be used as a high-resolution strain-gauge sensor.

## Results

### Flexible and stretchable photonic crystal nanolaser

**Figure 1:**
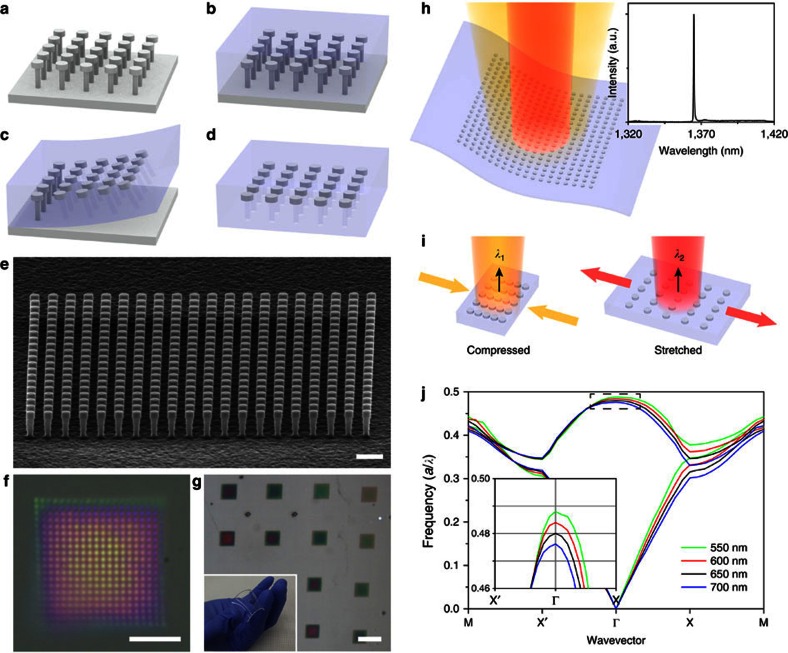
Flexible and stretchable nanolaser. (**a**–**d**) Schematic illustrations of fabrication process. A high-index semiconductor iron-nail-shaped rod array is fabricated (**a**) and embedded in an optically transparent low-index flexible polymer (**b**). The rod array is peeled off from the substrate (**c**), and the supporting posts are selectively removed by wet-etching (**d**). (**e**) Tilted SEM image of a square lattice InGaAsP iron-nail-shaped rod-type PhC structure before embedding it in PDMS. The lattice constant, radii of individual nail heads and supporting posts, and thicknesses of individual nail heads and supporting posts are 650, 200, 120, 250 and 800 nm, respectively. Scale bar, 1 μm. (**f**) Optical microscopy image of a PhC structure embedded deeply in PDMS. Scale bar, 5 μm. (**g**) Optical microscopy image of the array of PhC structures embedded in PDMS. Scale bar, 20 μm. Inset: a photograph showing the flexibility and transparency of the fabricated laser sample. (**h**) Schematic illustration of optically pumped lasing from a PhC structure embedded in a flexible and transparent substrate. Inset: measured single-mode lasing peak at a wavelength of 1,364.9 nm. (**i**) Schematic illustrations exhibiting the tuning mechanism of the lasing wavelength in stretched or compressed PhC laser devices. (**j**) Calculated transverse-electric-like photonic band diagrams of the PhC structures embedded in PDMS. The lattice constant is varied from 550 to 700 nm in one direction (the *x* direction), whereas the lattice constant in the other direction (the *y* direction) is fixed at 650 nm. The other structural parameters are the same as those in **e**. The *y* axis is the normalized frequency in units of *a*/*λ* and the *x* axis is the wavevector, where *a* is a fixed lattice constant of 650 nm and *λ* is the wavelength in free space. Inset: magnified diagram around the first Γ-point band-edge mode, where the *x* direction lattice constant is 550 (green), 600 (red), 650 (black) and 700 nm (blue).

The fabrication of our flexible and mechanically tunable strain-sensitive laser device involves several key steps (Fig. 1a–d; Methods). First, a high-index semiconductor iron-nail-shaped rod array structure serves as a high-quality PhC platform (Fig. 1a). Second, an optically transparent viscoelastic polymer material with low refractive index is utilized to uniformly cover the PhC structure (Fig. 1b). Third, the iron-nail-shaped rod array is peeled off from its supporting substrate when the flexible medium is separated from the substrate (Fig. 1c). Fourth, the posts of the iron-nail-shaped rods are selectively wet-etched to increase the optical confinement of the PhC structure and reduce optical loss through the posts (Fig. 1d). A scanning electron microscopy (SEM) image of the initial structure depicted in Fig. 1a shows the fabricated PhC structure, which consists of a 20 × 20 square lattice of iron-nail-shaped rods with a lattice constant of 650 nm (Fig. 1e). Figure 1f,g shows optical microscopy images of a single device (Fig. 1f) and an array (Fig. 1g) of the fabricated PhC devices with flexible polydimethylsiloxane (PDMS) as the surrounding material. No structural distortion or misalignment of the rods was observed (Fig. 1f), which demonstrates the robustness and reliability of our fabrication process. In addition, the zoomed out optical microscopy image of PhC devices with different structural parameters (Fig. 1g) demonstrates fabrication of a high yield and large-scale device array. The mechanical compressibility and stretchability of our device are also shown in the inset of Fig. 1g.

The schematic illustrations of Fig. 1h,i elucidate the basic working principle of the mechanically tunable strain-sensitive laser device. When the square lattice PhC structure embedded in a thin sheet of transparent and flexible PDMS is optically pumped, single-mode lasing is achieved from a resonant photonic band-edge mode (inset of Fig. 1h). Then, one can effectively control the resonant frequency of the band-edge mode by applying mechanical strain and causing a major structural modification of the PhC structure (Fig. 1i). For example, as the flexible polymer layer is compressed or stretched in one direction, the lattice constant of the PhC structure in that direction changes accordingly, which leads directly to the modification of the photonic band structure and thus results in the frequency shift of the resonant band-edge mode. To examine the validity of our device concept, we calculated the photonic band diagrams of the square lattice PhC structure embedded in a PDMS sheet with varying lattice constants from 550 to 700 nm along one direction (the *x* direction) (Fig. 1j). The lattice constant in the other direction (the *y* direction) was fixed to 650 nm. As anticipated, the overall photonic band structure was modified as the lattice constant changed or strain was applied, showing an increase (green and red lines) or decrease (blue line) in frequency with respect to the frequency with a lattice constant of 650 nm (black line). In particular, a gradual frequency shift was clearly observed in the Γ-point band-edge mode (inset of Fig. 1j) from which the lasing mode of our PhC laser device originated. This result reveals that the lasing wavelength of our device can be tuned by applying a systematic deformation of the structure, which enables the device to be used as an efficient nanophotonic strain-gauge sensor.

### Characterization of the strain-gauge laser sensor

**Figure 2:**
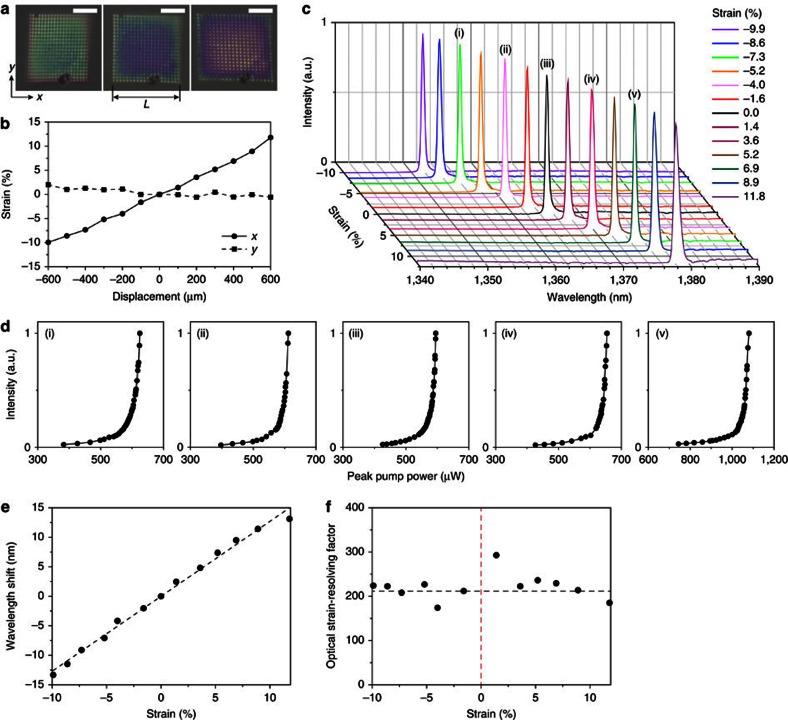
Spectroscopic characterization of the strain-gauge laser sensor. (**a**) Optical microscopy images of a fabricated PhC structure embedded in PDMS. Strains of −9.9% (left of panel), 0.0% (middle of panel) and 11.8% (right of panel) are applied along the *x* direction. Strain is defined as the percentage change of one side length (*L*) of the PhC structure. Scale bars, 5 μm. (**b**) Strains along the *x* (solid line) and *y* directions (dashed line) as a function of displacement of the sample stage in the *x* direction. (**c**) Measured lasing spectra of the strain-gauge PhC laser in **a** under different strains along the *x* direction. The applied strain varies from −10 to 12%. The PhC laser is optically pumped using a pulsed diode laser with a peak pump power of 700–1,200 μW. The linewidths of the lasing peaks are less than ∼0.6 nm for strains of ≤6.9%, and ∼0.8 nm and ∼0.9 nm for strains of 8.9% and 11.8%, respectively. (**d**) Measured output intensity as a function of the incident peak pump power under the strains in (i)–(v) in **c**. The lasing threshold powers are ∼600 (i), ∼590 (ii), ∼580 (iii), ∼640 (iv), and ∼1,050 μW (v). (**e**) The lasing wavelength shift in **c** with respect to that of the unstrained laser device is plotted as a function of the applied strain. The dotted line indicates a linear fit to the data. (**f**) The estimated optical strain-resolving factor is plotted as a function of the applied strain. The black and red dotted lines are the mean strain-resolving factor based on the strain sensitivity obtained in **e** and the experimental singularity line, respectively.

To characterize the mechanical flexibility and the induced structural strain of our strain-gauge laser device, we used a custom-built mechanical controller attached to a movable stage (Supplementary Fig. 1). As shown in three representative optical microscopy images in Fig. 2a, the PhC structure embedded firmly in PDMS was compressed (left of panel), pristine (middle of panel) or stretched (right of panel) in one direction (the *x* direction), showing discernible colour changes due to the structural variation. Stretching the sample causes an increase in the *x* direction lattice constant of the PhC structure, and is manifested as a red shift of the reflected wavelength^29^,^30^. No noticeable structural change was observed in the other direction (the *y* direction). Then, we systematically estimated the applied strain to the PhC structure by causing gradual mechanical deformation of the PDMS substrate repeatedly and by measuring the subsequent displacement of the PhC structure. The applied strains in the *x* and *y* directions were plotted as a function of displacement of the sample stage in the *x* direction (Fig. 2b). Here the strain is defined as the percentage change of one side length (*L* in Fig. 2a) of the PhC structure^31^. This graph shows a wide and monotonic change of strain from −10 up to 12% in the *x* direction, whereas the strain in the *y* direction remains almost unchanged. The mechanical deformation was repeatable and reversible, and thus the PDMS substrate was within the linear elasticity regime.

Next, we performed photoluminescence spectroscopy to examine the optical properties of our strain-gauge laser devices under different strains along the *x* direction. The devices were mounted on the same mechanical controller and optically pumped at room temperature using a 980-nm pulsed laser diode (10-ns pulses with 1% duty cycle). A × 40 microscope objective lens was used to focus the pumping beam to a spot size ∼3.6 μm, and the light emitted from the devices was fed into either an infrared linear array detector or an infrared camera using the same objective lens (see Methods; Supplementary Fig. 1). To assess the feasibility of a nanophotonic strain-gauge sensor, we systematically increased or decreased the applied strain and monitored the subsequent lasing wavelengths (Fig. 2c). The measurements exhibited several notable features. First, we observed a monotonic linear red shift (blue shift) of the laser peak when we gradually applied the positive (negative) strains up to 11.8% (−9.9%). The resonance shift can thus be used as an excellent measure of the change in applied strain, supporting the device concept in Fig. 1i,j. Second, our device demonstrated a maximum laser peak shift of 13 nm (−13 nm) for a positive (negative) strain of 11.8% (−9.9%). This reversible, wide-bandwidth and deterministic resonance wavelength tuning is distinguishable from previous work using local temperature tuning^26^,^27^, electro-optic modulation through free-carrier implantation^32^ and gas condensation^33^,^34^. Whereas there have been practical limits due to either irreversibility or the narrow range (less than a few nanometres) of wavelength tuning, our PhC laser devices embedded deeply in PDMS possess both reversible control and a wide range of wavelength tuning (up to ∼26 nm) under positive and negative strains. Third, the highly sharp single-mode lasing peak with a spectral linewidth of less than ∼0.6 nm provides a simple and stable tool for high-resolution sensing of strain. One can avoid potential difficulties originating from modal competition and intensity variation between multiple resonant peaks under different strains and excitation schemes^31^.

**Figure 3:**
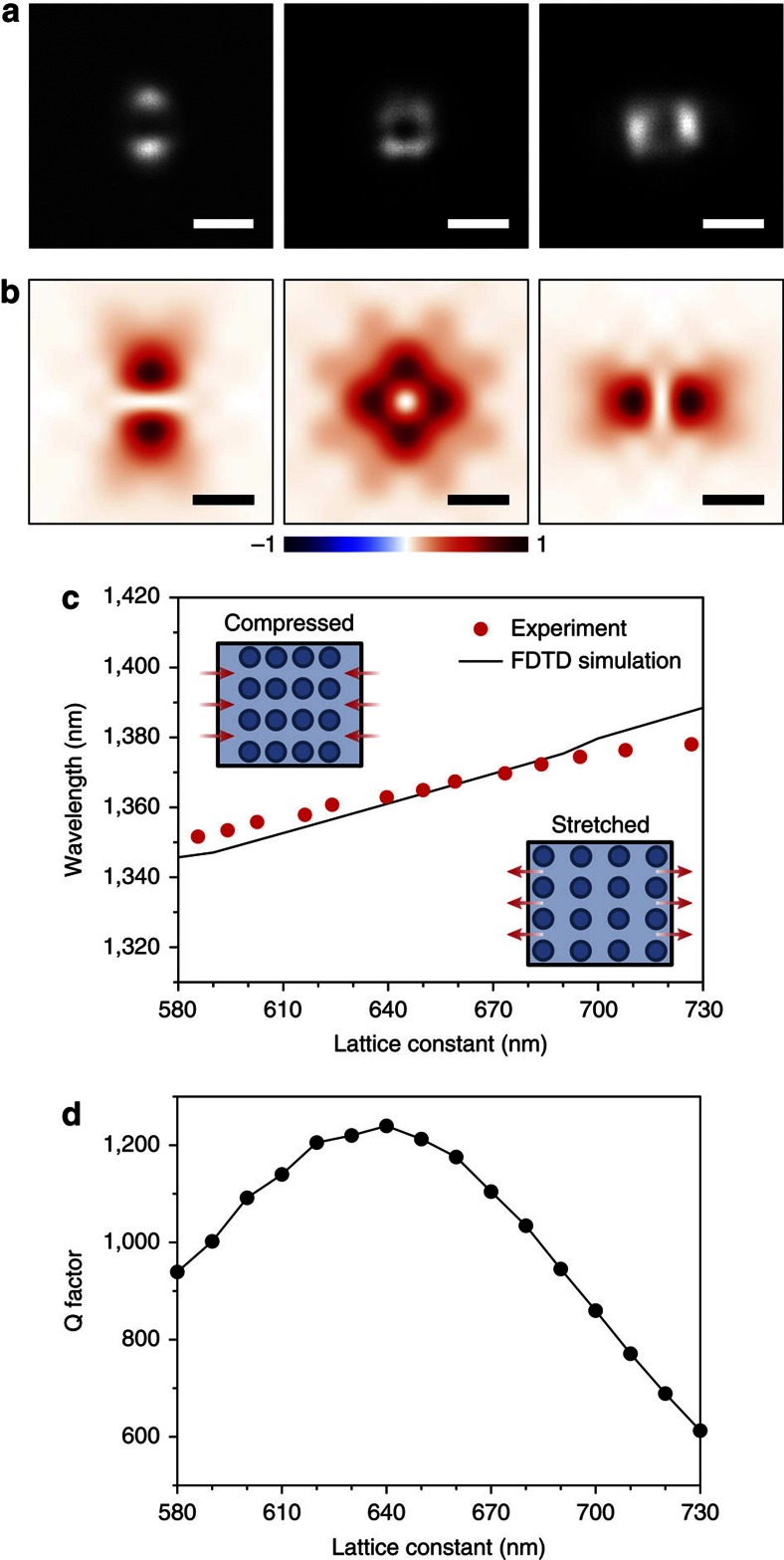
Visualization of applied strains and comparison with numerical simulations. (**a**) Measured visual responses of the lasing mode under different strains of −7.3% (left of panel), 0.0% (middle of panel) and 6.9% (right of panel) along the *x* direction. Scale bars, 5 μm. (**b**) Calculated *z*-components of the time-averaged Poynting vectors at a position 15 μm above the PhC structure. Lattice constants of 600 (left of panel), 650 (middle of panel) and 700 nm (right of panel) are used in the simulation, which give strains similar to those in **a**: lattice constants of 600, 650 and 700 nm correspond to strains of −7.7%, 0.0% and 7.7%, respectively. Scale bars, 5 μm. (**c**) Measured (red dots) and calculated (black line) resonant wavelengths plotted as a function of the *x* direction lattice constant of PhC structure. The measured wavelengths are from Fig. 2c. (**d**) Calculated *Q*-factors as a function of the *x* direction lattice constant. The *y* direction lattice constant is fixed at 650 nm.

We also characterized the laser properties by measuring the output peak intensity as a function of the incident peak pump power. Figure 2d shows five representative characteristic curves measured from the laser device in Fig. 2c under different strain conditions ((i) −7.3%, (ii) −4.0%, (iii) 0.0%, (iv) 3.6% and (v) 6.9%). Superlinear increases in output intensity are clearly observed in all cases. The lasing threshold of the unstrained device is ∼580 μW (Fig. 2d; (iii)). Whereas the lasing threshold slightly increased for the negative strains (Fig. 2d; (i), (ii)), a noticeable increase in threshold was observed for the positive strains (Fig. 2d; (iv), (v)). This asymmetric change in threshold is mainly due to strain-dependent quality (*Q*) factors of the Γ-point band-edge mode, which are analysed in Fig. 3d in more detail. Next, to investigate the resonance stability of the strain-gauge laser device, we measured the lasing wavelengths under the strains in (i)–(v) while increasing the peak pump power above the threshold (Supplementary Fig. 2). We clearly observed that the lasing wavelength under each applied strain remained unchanged in the above-threshold pumping condition. This result supports that the wavelength shift originates solely from the applied strain, not a local thermal effect or resonance instability caused by high pumping power.

In addition, the resonant wavelength shift (Δ*λ*=*λ*_strain_−*λ*_0_) of the lasing mode was plotted as a function of the applied strain (Fig. 2e). As expected, the graph shows an excellent linear relation between the wavelength shifts and strains and yields a corresponding optical strain sensitivity of ∼0.127 pm per μ*ɛ*, where *ɛ* denotes the applied strain. To assess the ability of an optical strain-gauge sensor to quantify spectrally resolvable strain, we define a physical parameter that includes the strain sensitivity and spectral resolution in the optical system, which is called the ‘optical strain-resolving factor'. The optical strain-resolving factor is defined as (Δ*λ*/*ɛ*)/δ*λ*, where δ*λ* is the full width at half maximum spectral linewidth, that is, the ratio of a resonant wavelength shift in an applied strain to a spectral resolution of the resonance. In Fig. 2f, the optical strain-resolving factor was estimated using the resolution-limited spectral linewidth (δ*λ*=0.6 nm) in our measurement system and plotted as a function of the applied strain. For comparison, we obtained the mean strain-resolving factor directly from the strain sensitivity in Fig. 2e (black dotted line). The estimated optical strain-resolving factors are in the range of 210±20, which is relatively large because of the sub-nanometre spectral linewidth (Supplementary Methods). Consequently, a minimum strain of 0.5% is spectrally resolvable in our optical system.

### Visualization of applied strains

To further examine the lasing modes of our strain-gauge laser device, we measured the lasing mode images under different strain conditions and compared them with numerical simulations. In Fig. 3a, three representative lasing mode images were captured from the device in Fig. 2 under negative (−7.3%, left of panel), pristine (0.0%, middle of panel) or positive (6.9%, right of panel) strains along the *x* direction. A donut-shaped mode image with a central intensity node was observed in the unstrained device^23^. As positive (negative) strain was applied, the lasing mode instantaneously changed to a dipole-shaped image with an intensity minimum along the direction perpendicular (parallel) to the strain. The lasing mode also changed to be linearly polarized along the *x* and *y* directions in the left and right images in Fig. 3a, respectively (Supplementary Fig. 3). Thus, the visual response of different signs of strain, which was a result of structural and the corresponding modal symmetry breaking under positive or negative strains, was demonstrated for the first time in a strain-gauge optical system, to the best of our knowledge. Three-dimensional (3D) finite-difference time-domain (FDTD) simulations agreed well with this experimental observation (Fig. 3b). We used the same structural parameters as those in Fig. 3a and calculated the *z*-component of time-averaged Poynting vector distributions of the Γ-point band-edge mode at a position 15 μm above the PhC structure. Lattice constants of 600 (left of panel), 650 (middle of panel) and 700 nm (right of panel) in Fig. 3b correspond to negative (−7.7%), pristine (0.0%) and positive (7.7%) strains, respectively, and thus we can directly compare these simulation results with the measured images in Fig. 3a. The excellent agreement between the measured and simulated results supports the strain-dependent lasing mode images in our laser device. In addition, the calculated *x* and *y* components of electric fields are useful to understand the shapes of the lasing modes under strain conditions (Supplementary Fig. 4).

3D FDTD simulations also reproduced well the other experimental observations in Fig. 2, including lasing wavelengths and threshold pump powers. We changed the lattice constant of the PhC structure from 580 to 730 nm with a step of 10 nm along the *x* direction, which corresponds to strains from −10.8 to 12.3%. The calculated resonant wavelengths and *Q*-factors of the Γ-point band-edge mode were plotted as a function of the lattice constant (Fig. 3c,d). The resonant wavelengths in Fig. 3c agree well with the measured lasing wavelengths in Fig. 2c, supporting our mode analysis of the Γ-point band-edge mode. In addition, the calculated *Q*-factors in Fig. 3d can explain unambiguously the asymmetric strain-dependent change in threshold in Fig. 2d. For example, the *Q*-factors decreased significantly for increasing positive strains, similar to the measured threshold values. However, the relatively small decrease in the *Q*-factors for the negative strains, due to a blue shift of the resonant wavelength and an increase in the effective thickness, supports a small change in lasing threshold.

### Strain-based tunable laser pH sensor

**Figure 4:**
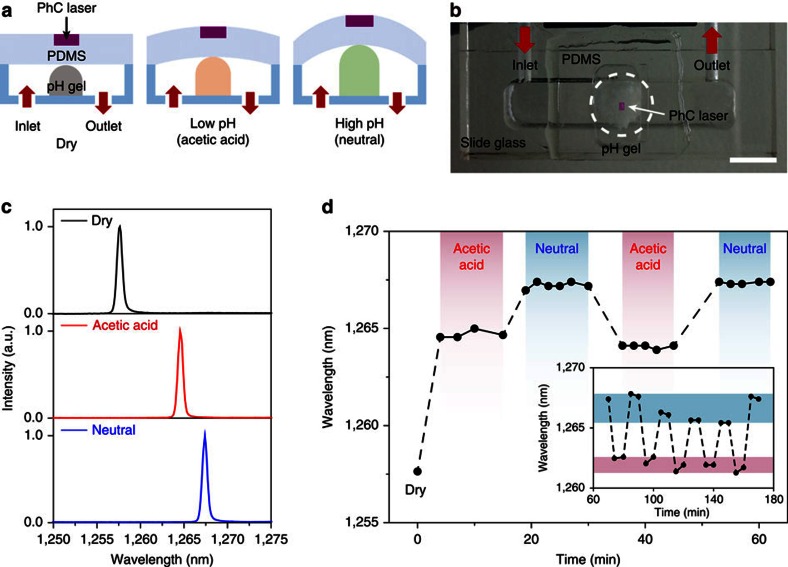
Mechanical strain-based tunable laser pH sensor. (**a**) Schematic illustrations showing the working principle of pH sensing using the laser device. An aqueous solution with a given pH level is injected into the fluidic channel and meets the pH-sensitive hydrogel in the chamber. Different mechanical strains are applied to the strain-sensitive flexible laser device on top of the chamber, depending on the volume change of the pH-sensitive hydrogel. (**b**) A photograph of the opto-fluidic pH sensor that includes a strain-sensitive flexible PhC laser device, the pH-sensitive hydrogel, and the transparent plastic fluidic channels. Scale bar, 1 cm. (**c**) Measured lasing spectra of an optically pumped strain-sensitive flexible PhC laser in dry conditions (no solution; top of panel), acetic acid (pH 2.5; middle of panel) and a neutral solution (pH 7.0; bottom of panel). The incident pump power is 700–800 μW, and the lasing wavelengths are 1,257.6 (top of panel), 1,264.6 (middle of panel) and 1,267.4 nm (bottom of panel). (**d**) Measurement of the reversible repeatability and temporal stability of the pH sensor of **c**. The spectral behaviour of lasing wavelength was monitored over time while the pH level in the aqueous solution was reversibly and repeatedly switched between acetic acid (red) and neutral (blue) solutions. The measured lasing wavelengths are 1,264.7±0.2 nm (4–15 min; red), 1,267.2±0.2 nm (19–30 min; blue), 1,264.1±0.1 nm (36–45 min; red) and 1,267.4±0.1 nm (53–62 min; blue). Inset: multi-cycle test over longer time.

To take full advantage of the strain-sensitive properties of the laser device, we designed a simple opto-fluidic system and performed a mechanical strain-based chemical-sensing experiment (Fig. 4a). The PhC laser device embedded in flexible PDMS is placed on top of a fluidic chamber that consists of inlet and outlet channels and pH-sensitive hydrogel (Supplementary Fig. 5). The hydrogel offers reversible and tunable volume changes for different pH levels, and the resultant volume of the hydrogel increases with the pH value. When aqueous solutions with different pH values are injected into the fluidic chamber, the changes in the volume of the hydrogel lead to different strains on the PhC laser device on top and give rise to different lasing wavelength shifts (Fig. 4a): the measurement of the lasing wavelength shift ultimately enables pH sensing. Figure 4b shows an optical image of the PhC laser device attached to the transparent fluidic chamber. Then, laser spectra were measured under three different pH conditions (Fig. 4c): dry state (top of panel), acetic acid (middle of panel, pH 2.5) and neutral solutions (bottom of panel, pH 7.0). Initially, the PhC device was optically pumped when the hydrogel was in the dry state (Fig. 4c; top of panel). Single-mode lasing was observed at a wavelength of ∼1,257.6 nm. Next, when the low pH (acetic acid) solution was injected into the chamber, the hydrogel started to swell. In the steady state, the lasing wavelength was red-shifted to ∼1,264.6 nm because of the volume change in the hydrogel (Fig. 4c; middle of panel). We further increased the pH level by buffering the solution at pH 7. A greater red shift to ∼1,267.4 nm was successfully measured in the steady state (Fig. 4c; bottom of panel).

Furthermore, we examined the reversible repeatability and temporal stability of our strain-based tunable laser pH sensor (Fig. 4d). The photoluminescence measurement was first performed in the dry state. Then, we repeatedly changed the pH level of the aqueous solution in the fluidic chamber between the acetic acid and neutral solutions, and monitored the spectral behaviour of the lasing wavelength over time. The experimental results in Fig. 4d exhibit several features. First, excellent spectral stability was demonstrated for each pH level with spectral s.d.'s of ±0.2 nm (first acetic acid solution), ±0.1 nm (second acetic acid solution), ±0.2 nm (first neutral solution) and ±0.1 nm (second neutral solution). Second, a deterministic differentiation of the pH level was achieved during the reversible and repeated measurements over time (<60 min). Third, our strain-based pH sensor worked well over longer time (>180 min), although a gradual degradation in the spectral stability was observed because of the limited reconfigurability of the pH-sensitive hydrogel (inset of Fig. 4d). Taken together, the strain-gauge laser device functioned as a sensitive strain-based pH sensor. Furthermore, our laser device was in an attachable and flexible thin substrate, which can enable potential applications in chemical and biological systems such as temporal recording or the mapping of local surface strains on living cells.

## Discussion

In summary, we have demonstrated a strain-gauge nanolaser by integrating an iron-nail-shaped rod-type PhC platform with a flexible and transparent polymer substrate. Systematic control of the mechanical strain allows the manipulation of the photonic band structure, and the subsequent spectral behaviour of the single-mode lasing peak serves as a strain-gauge mechanism. Photoluminescence measurements showed that a wide range of wavelength tuning (up to ∼26 nm) with a sub-nanometre scale spectral resolution (<∼0.6 nm) was achieved under repeatable and reversible positive (negative) strains of 11.8% (−9.9%). In addition, full 3D FDTD simulations unambiguously reproduced the experimental observations, such as the lasing mode images and resonant wavelength shift, and supported our quantitative analysis of the lasing threshold and strain sensitivity. We also demonstrated a locally interactive strain-based chemical pH sensor, showing robust reversible repeatability and temporal stability. Our technological approach that uses a band-edge lasing mode in the defect-free PhC structure enables detecting nanoscale alternations in arbitrary positions of the structure through the changes of lasing wavelength, and consequently it should be feasible to map local surface strains and deformations in many structures. Furthermore, the strain-gauge nanolaser or an array may be useful to deterministically differentiate particular chemical species and their shapes and concentrations in various chemical/biological systems.

## Methods

### Device fabrication

We defined 20 × 20 square-lattice rod arrays with a lattice constant of 650 nm on a 250-nm-thick InGaAsP/800-nm-thick InP/100-nm-thick InGaAs/InP substrate wafer using electron-beam lithography and chemically assisted ion-beam etching. The InGaAsP slab included three quantum wells with a central emission wavelength of ∼1.3 μm. The 800-nm-thick InP layer was selectively and partially wet-etched using a diluted HCl:H_2_O (3:1) solution to form iron-nail-shaped rod array. The etching time was ∼20 s at 5 °C. Then, the InGaAsP/InP iron-nail-shaped rod arrays were covered with a thin, transparent and flexible PDMS sheet, and were subsequently detached from the substrate by wet-etching of the InP layer during few minutes. The fabrication was finalized by the complete wet-etching of the InP layer.

### Optical measurement

The flexible PDMS samples with PhC laser devices were mounted and fixed to a customized digital controller that consisted of a micrometre and a single-axis translation stage. The controller was used to stretch or compress the flexible samples (Supplementary Fig. 1). In Figs 2 and 3, a 980-nm pulsed laser diode (10-ns pulses with 1% duty cycle) was used to optically pump the PhC laser devices at room temperature. The light emitted from the laser devices was collected by a × 40 microscope objective lens with a numerical aperture of 0.55 and focused onto either an infrared one-dimensional array detector (PyLoN, Princeton Instruments) or an InGaAs infrared camera (C10633, Hamamatsu).

### pH-sensing experiment

The opto-fluidic system for the pH-sensing experiment consisted of a customized transparent plastic plate that included inlet and outlet channels, pH-sensitive hydrogel (AquaGel pH, Akina Incorporated) and a thin PDMS sheet with the PhC laser devices. The hydrogel offered reversible and tunable volume changes that could be specifically sensitized to pH levels, and the resultant volume of the hydrogel increased with the pH value. The flexible PDMS sheet with the laser devices was placed on top of the pH-sensitive hydrogel (Supplementary Fig. 5). In Fig. 4, an acetic acid solution at pH 2.5 and a neutral buffer solution at pH 7.0 were reversibly and repeatedly injected into the fluidic chamber using an automated syringe pump. The photoluminescence measurement was performed using the optical setup in Fig. 2.

### Numerical simulations

3D FDTD simulations were performed to calculate the photonic band diagrams (Fig. 1j), time-averaged Poynting vectors (Fig. 3b; Supplementary Fig. 3c), resonant wavelengths (Fig. 3c), *Q*-factors (Fig. 3d) and near-field mode profiles (Supplementary Fig. 4). We used the structural parameters obtained from the SEM image in Fig. 1e. The refractive indices of InGaAsP and PDMS were set to 3.30 and 1.42, respectively. For the calculation of photonic band diagrams, a periodic boundary condition was applied along the *x* and *y* directions, and a perfectly matched layer was applied along the *z* direction. For the other calculations, perfectly matched layers were applied at all boundaries of the calculation domain with a spatial resolution of 10 nm. The calculation domain sizes were 20.0 × 20.0 × 17.0 μm^3^ for the calculation of time-averaged Poynting vectors and 12.0 × 12.0 × 3.5 μm^3^ to calculate the resonant wavelengths, *Q*-factors and near-field mode profiles.

## Additional information

**How to cite this article:** Choi, J.-H. *et al*. A high-resolution strain-gauge nanolaser. *Nat. Commun.* 7:11569 doi: 10.1038/ncomms11569 (2016).

## Supplementary Material

Supplementary Information Supplementary Figures 1-5 and Supplementary Methods
