# Supplementary material for: A high-resolution strain-gauge nanolaser
Source: Nat Commun. 2016 May 12;7:11569. doi: 10.1038/ncomms11569 (PMC4865857; doi:10.1038/ncomms11569)
Supplement: Supplementary Information — Supplementary Figures 1-5 and Supplementary Methods [file ncomms11569-s1.pdf]

Supplementary Figures

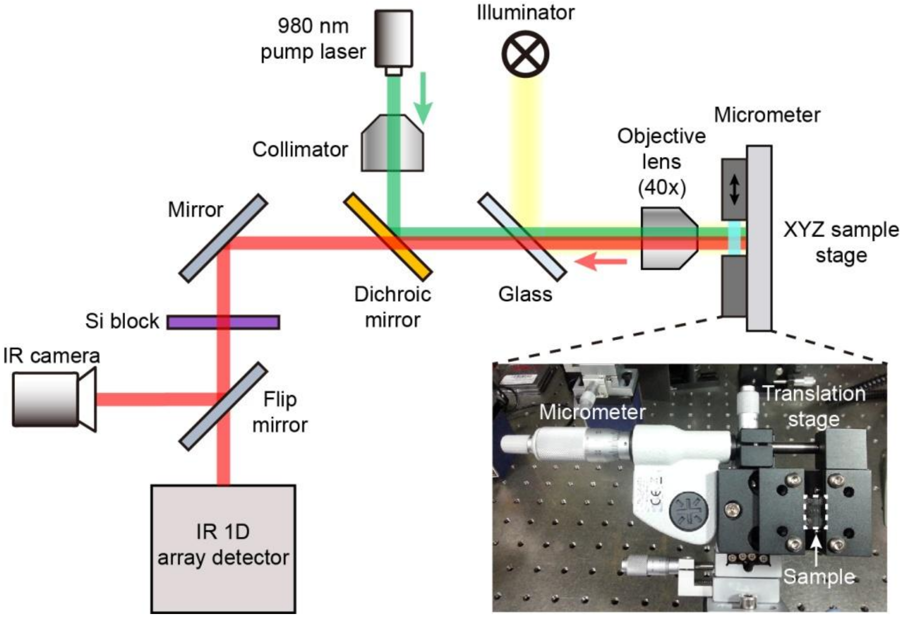

Supplementary Figure 1 | Optical measurement setup. Our flexible PhC laser samples were mounted on a customized digital controller (micrometer + single-axis translation stage) attached to the XYZ translation stage (see photograph). The controller was used to apply positive (stretched) or negative (compressed) strains on the sample (white dotted square). In addition, the samples were optically pumped at room temperature using a 980-nm pulsed laser diode (10-ns pulses with 1% duty cycle). A 40× microscope objective lens with a numerical aperture of 0.55 was used to focus the pumping beam and collect the light emission from the devices.

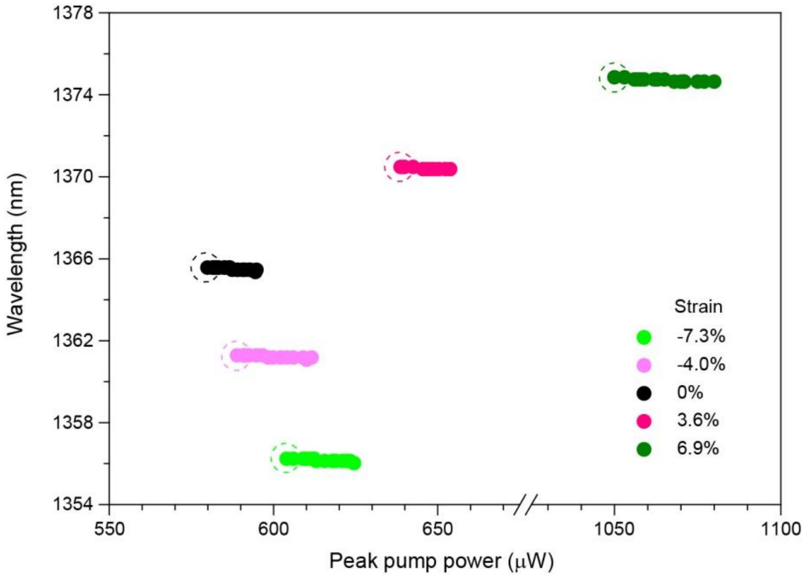

Supplementary Figure 2 | Spectral stability of lasing wavelength at above-threshold pump powers. Measured above-threshold wavelengths are plotted as a function of the incident peak pump power under the strain conditions in Fig. 2d; -7.3% (green), -4.0% (pink), 0% (black), 3.6% (magenta), and 6.9% (olive). Each dashed circle indicates the threshold pump power under the strain. We observe no noticeable change in the above-threshold wavelength with increasing pump power under any strain. This result demonstrates that the thermal effect is negligible in our laser device.

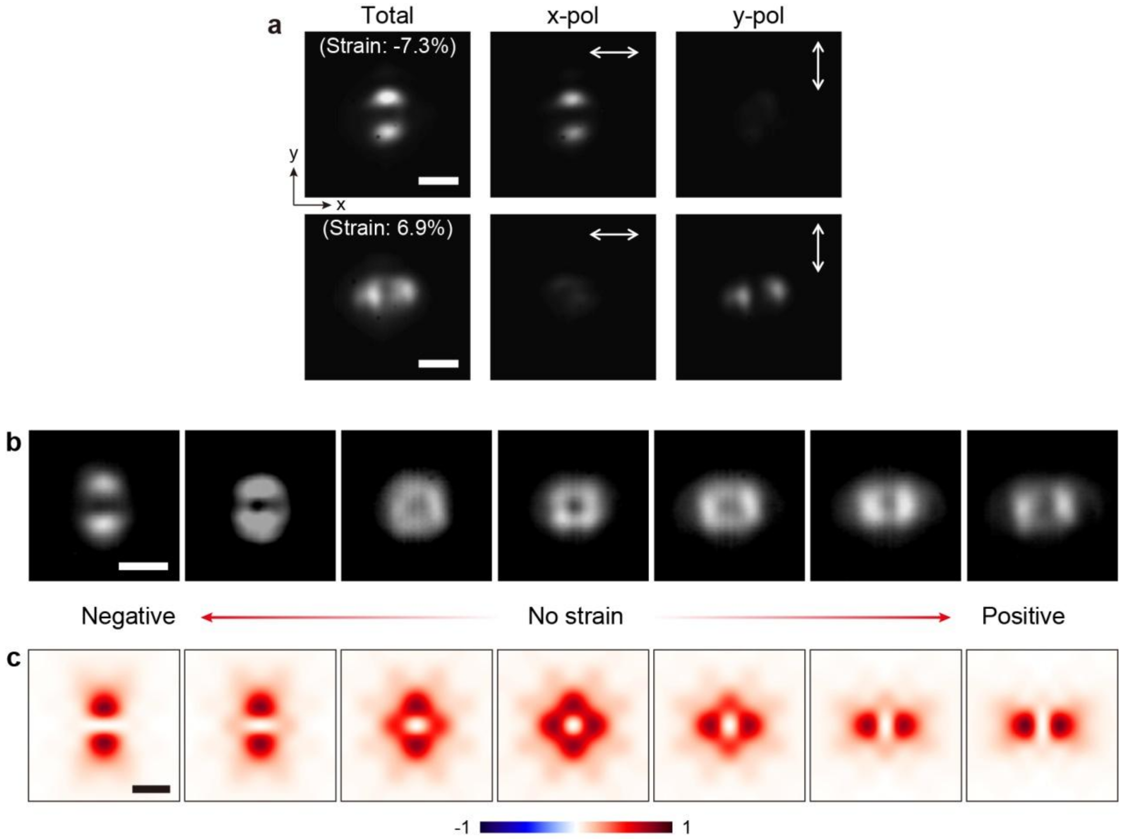

Supplementary Figure 3 | Measurement and simulation of lasing mode images. (**a**) Measured total (left) and polarization-resolved (middle and right) lasing mode images under negative (top; -7.3%) and positive (bottom; 6.9%) strain conditions. A linear polarizer was positioned in front of the IR camera for the measurement of the polarization-resolved images. The lasing mode is linearly polarized along the *x*-direction (*y*-direction) as the negative (positive) strain is applied. (**b**) Measured lasing mode images under negative and positive strain conditions (left to right; -7.3%, -5.2%, -1.6%, 0%, 1.4%, 5.2%, and 6.9%). (**c**) Calculated mode images under negative and positive strain conditions (left to right; -7.7%, -4.6%, -1.5%, 0%, 1.5%, 4.6%, and 7.7%). The *z*-components of the time-averaged Poynting vectors were calculated using the same method as the one in Fig. 3b. The scale bars are all 5 μm.

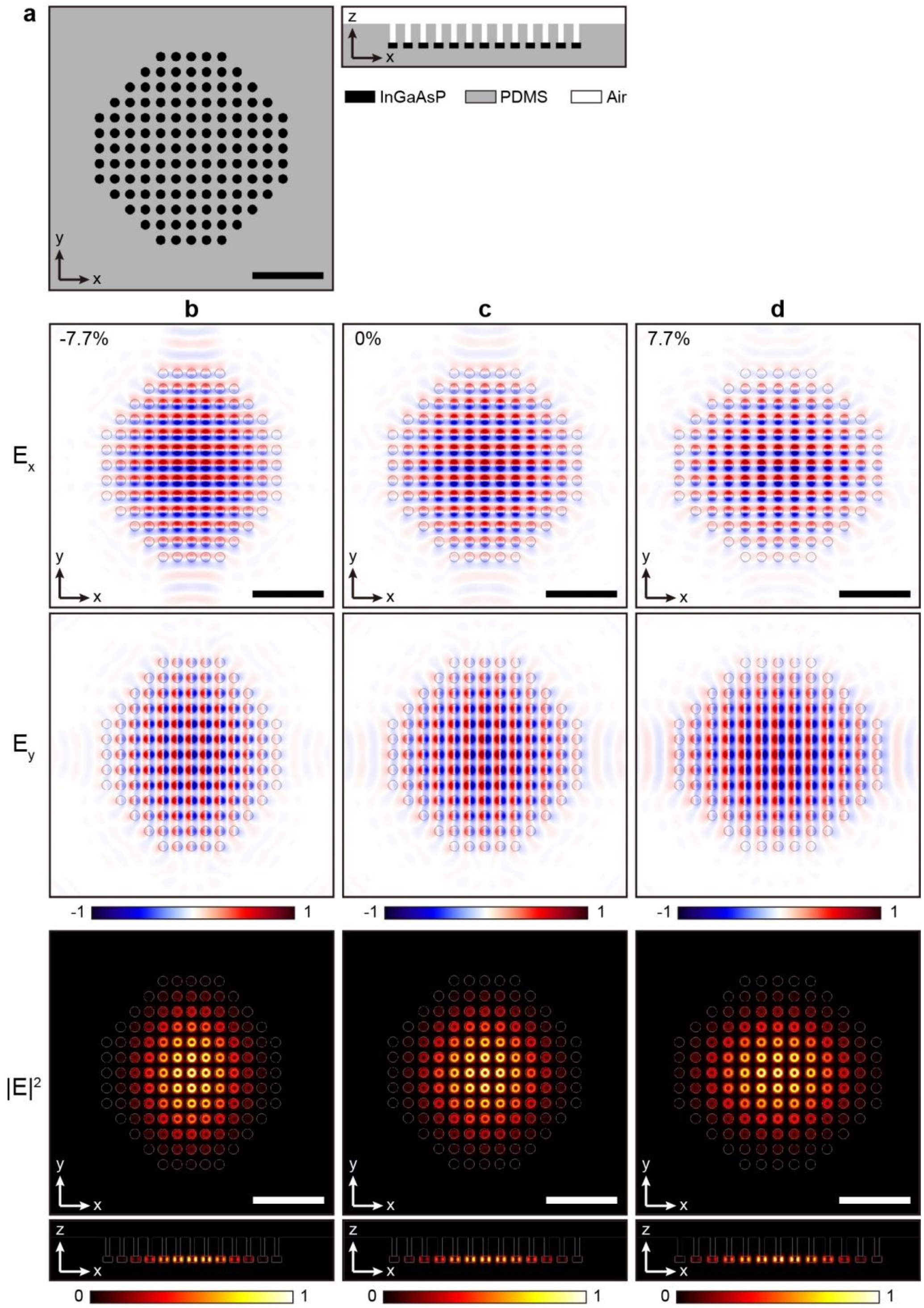

Supplementary Figure 4 | Calculated near-field band-edge mode profiles. (**a**) Top-view and side-view simulation structures. (**b**)-(**d**) Calculated E_x and E_y field profiles and electric-field intensities (top to bottom) under negative (**b**), pristine (**c**), and positive (**d**) strains. The lattice constants and corresponding strains in the *x*-direction are as follows: (**a**) (600 nm, -7.7%), (**b**)

(650 nm, 0%), and (**c**) (700 nm, 7.7%). The lattice constants in the *y*-direction are 650 nm. The E_x and E_y fields were normalized by the maximum amplitude of the E_x field in (**b**) or by the maximum amplitude of the E_y field in (**d**). We considered an effective pumping area with a diameter of ~7.2 μm. All the scale bars are 5 μm.

The E_x and E_y fields can be considered effectively as two opposite-phase dipoles oscillating along the *x*- and *y*-directions, respectively. Under pristine strain, all dipoles in the E_x and E_y fields have the same amplitudes and thus, a donut-shaped mode profile with a central intensity node is generated (Fig. 3b; middle). Under negative (positive) strain, a dipole-shaped mode profile is shown because the amplitudes of the dipoles in the E_x (E_y) field are stronger than the ones in the E_y (E_x) field (Fig. 3b; left and right).

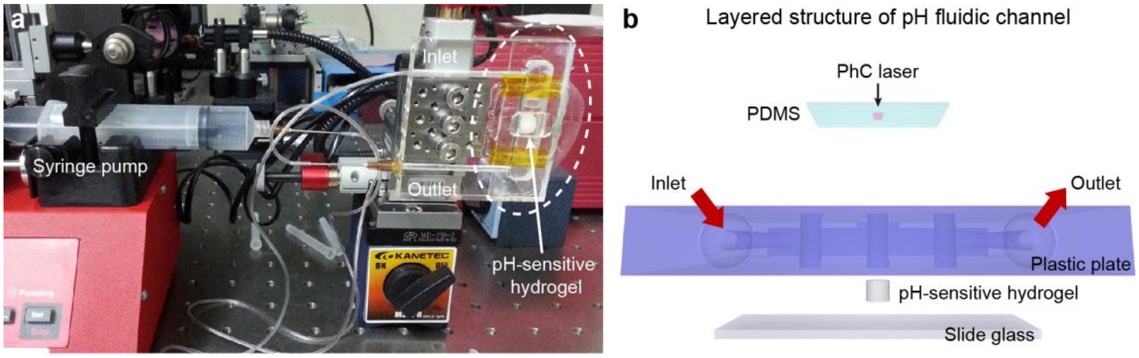

Supplementary Figure 5 | Mechanical strain-based pH sensing experimental setup and schematic layout of the opto-fluidic system. (**a**) A photograph showing the custom-built opto-fluidic channel and solution-injection system. (**b**) A schematic of the opto-fluidic system. A transparent plastic body consists of inlet and outlet fluidic channels and a central chamber. The thin PDMS sheet with the PhC laser device is placed on top of the fluidic chamber. The pH-sensitive hydrogel on the slide glass is located underneath the PhC laser device.

Supplementary Methods

Optical strain-resolving factor. The optical strain-resolving factor is defined as $(\Delta\lambda/\varepsilon)/\delta\lambda$, where $\Delta\lambda$, ε, and $\delta\lambda$ are the resonant wavelength shift compared to the unstrained case, the applied strain, and the spectral resolution of the resonance, respectively. This parameter provides an efficient and quantitative measure to understand how precisely one can spectrally resolve strains using an optical resonance in the system. For example, if the resonance shift is small ($\Delta\lambda$ = small) for a given strain, the optical strain-resolving factor is small, which shows that the strain is not easily distinguishable spectrally. However, if the optical resonance in the system is sharp and of high quality ($\delta\lambda$ = small) for a given strain, the optical strain-resolving factor is large, and thus, the strain can be clearly distinguished.
